# Half-dose Alteplase for Sub-massive Pulmonary Embolism Directed by Emergency Department Point-of-care Ultrasound

**DOI:** 10.5811/westjem.2014.12.24130

**Published:** 2015-01-12

**Authors:** Richard Amini, Ashish R. Panchal, David Bahner, Srikar Adhikari

**Affiliations:** * University of Arizona, Department of Emergency Medicine, Tucson, Arizona; † Ohio State University, Department of Emergency Medicine, Columbus Ohio

## Abstract

This report describes a patient with sub-massive pulmonary embolism (PE) who was successfully treated with half-dose thrombolytics guided by the use of point-of-care (POC) ultrasound. In this case, POC ultrasound was the only possible imaging since computed tomography was contraindicated. POC ultrasound demonstrated a deep vein thrombosis and evidence of cardiac strain. In situations or locations where definitive imaging is unobtainable, POC ultrasound can help diagnose submassive PE and direct the use of half-dose tissue plasminogen activator.

## INTRODUCTION

Pulmonary embolus (PE) is a life-threatening condition affecting 250,000 people annually.^1^ In the emergency department (ED), computed tomography angiography (CTA) is the method of choice to diagnose PE; however, it may not be feasible to obtain a CTA when patients have abnormal kidney function, hemodynamic instability, or are in resource-limited areas. In these situations, thrombolytic treatment may be delayed or withheld due to risk benefit concerns. Although thrombolytics decreases clot burden faster than heparin, the complication of intracerebral hemorrhage has led to hesitant physician use. Half-dose tissue plasminogen activator (tPA) research demonstrates decreased complications with similar clot resolution; however, its true influence on morbidity and mortality remains unknown.^2^

### Case Report

A patient presented to the ED with a chief complaint of sudden onset diaphoresis, dyspnea and near-syncope with minimal exertion. Aside from recent travel, the patient denied any other medical or surgical history. Triage vital signs were temperature 36.3°C, blood pressure 126/83mm Hg, heart rate 123 beats/min, respiratory rate 34 breaths/min, SpO2 85% on room air and required a non-rebreather to maintain oxygen saturation above 95%. Initial impression was a dyspneic patient without respiratory distress. Cardiac exam demonstrated tachycardia with irregular rate, and his lung exam was normal. Extremity evaluation demonstrated a slightly enlarged right lower extremity with trace edema. The remainder of the physical examination was normal.

**Figure 1:**
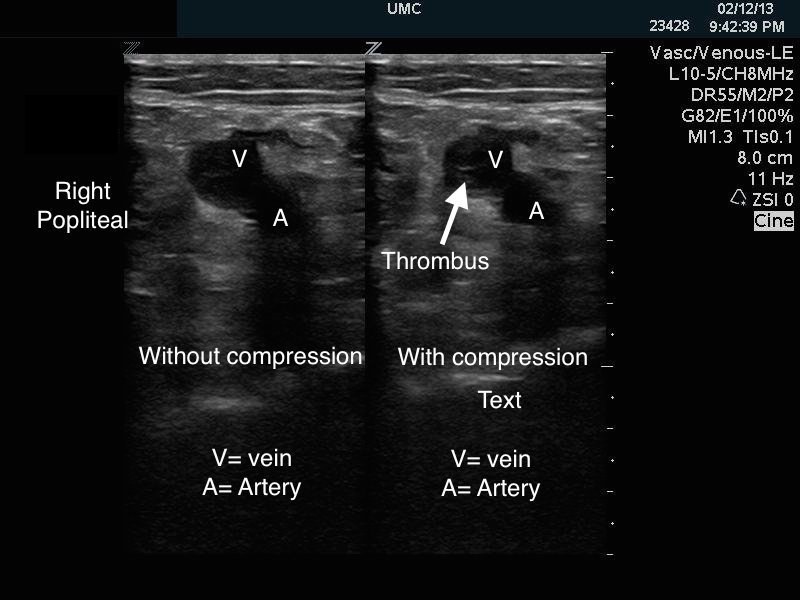
Dual image demonstrating right lower extremity popliteal vein clot with and without compression. The popliteal vein does not compress demonstrating active popliteal deep vein thrombosis.

**Figure 2:**
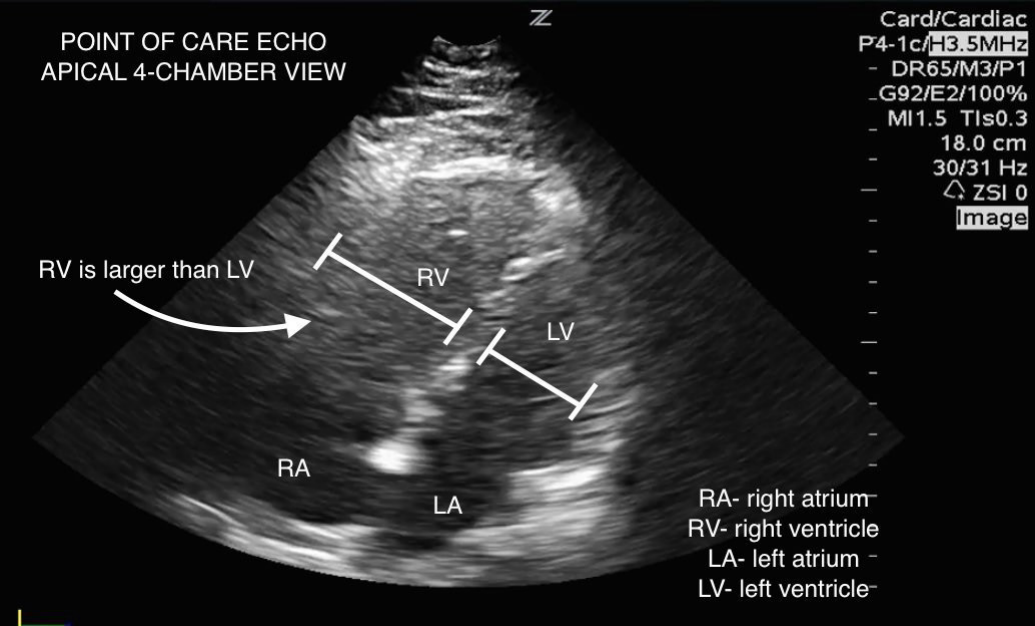
Point-of-care echocardiogram in apical 4-chamber view demonstrates significant right ventricle dilation.

Initial electrocardiogram (ECG) displayed atrial fibrillation with rapid ventricular response (124 beats/min); no evidence of ischemia, infarction or patterns concerning for right ventricle strain. The patient also had an elevated Troponin (1.8ng/ml) and B-type Natriuretic Peptide (888pg/ml). Chest radiograph was normal, but definitive CTA imaging to diagnose PE could not be pursued due to an elevated creatinine level (1.8mg/dl). Point-of-care (POC) ultrasound of the right lower extremity demonstrated a femoral vein deep vein thrombosis (DVT) extending from the sapheno-femoral junction to the popliteal fossa (Figure 1). POC cardiac ultrasound revealed right heart strain (dilatation, hypokinesis, and paradoxical septal motion) and a plethoric inferior vena cava (Figure 2). Given these findings, the patient was diagnosed with a sub-massive PE.

Although this patient was not hemodynamically unstable, it was clear that the patient could decompensate at any moment. The decision was made to start half-dose tPA with heparin infusion.^2^ One hour into the infusion, the patient appeared more comfortable, with improved vital signs: blood pressure 123/83mm Hg, heart rate 95 beats/min, respiratory rate 20 breaths/min, and SpO2 of 100% on a non-rebreather. The patient’s atrial fibrillation resolved without any dysrhythmic agents. Eight hours later, the patient was asymptomatic and no longer required oxygen.

After resolution of the patient’s acute renal injury, a CTA was performed, which demonstrated extensive PEs in all five pulmonary arteries as well as possible early infarct of the left lower lung. The patient was transitioned to subcutaneous low molecular weight heparin and warfarin and was discharged 72 hours after presentation.

## DISCUSSION

Sub-massive PE is considered in hypoxic and dyspneic patients who are hemodynamically stable but demonstrate signs of heart strain as seen by ECG changes, cardiac biomarkers, or sonographic evidence of right ventricular strain.^3^ Treatment with thrombolytics may prevent clinical deterioration and morbidity; however, it is associated with increased risk of intracranial hemorrhage.^4^ Some experts choose not to thrombolyse these patients because there is insufficient evidence for reducing mortality.^5^ New research demonstrates that treating sub-massive PEs with half-dose tPA may be safer and decrease long-term complications such as pulmonary hypertension.^2^ Despite this, emergency physicians must be confident in their diagnosis of PE with POC ultrasound prior to making this decision.

In this case, POC ultrasound was performed by an emergency ultrasound fellowship-trained physician. First, POC ultrasound helped diagnose a lower extremity DVT, which carries concomitant PE in 40–50% of cases.^6^,^7^ When the patient could not undergo definitive imaging secondary to acute renal injury, POC ultrasound demonstrated a dilated right heart, decreased right ventricular contractility, interventricular septal wall motion irregularity, and a plethoric inferior vena cava (Video). Although each of these findings are not sufficient for the diagnosis of PE, a recent study by Nazerian et al. demonstrated a sensitivity of 90% and a specificity of 86.2% when imaging the heart, lung, and veins.^8^ In this case, POC ultrasound confirmed the diagnosis of sub-massive PE and directed treatment with half-dose tPA in addition to systemic heparin.

While administration of thrombolytic therapy is controversial, and outside the scope of this article, POC ultrasound can assist in the decision making process. Due to the expanded use of POC ultrasound in emergency medicine (EM) and increased emphasis on ultrasound training in EM residency programs, it is reasonable to believe that all graduating EM residents will have the skills necessary to make this diagnosis.^9^ POC ultrasound can assist with rapid evaluation and treatment of patients with suspected sub-massive PE, especially in patients with contraindications to CTA.

## Figures and Tables

**Video:** Initial POC ultrasound.
